# Non-Contact Screening of OSAHS Using Multi-Feature Snore Segmentation and Deep Learning

**DOI:** 10.3390/s25175483

**Published:** 2025-09-03

**Authors:** Xi Xu, Yinghua Gan, Xinpan Yuan, Ying Cheng, Lanqi Zhou

**Affiliations:** 1 Hunan Provincial Key Laboratory of Intelligent Information Perception and Processing Technology, Hunan University of Technology, Zhuzhou 412007, China; xebox@sohu.com; 2 School of Computer Science and Artificial Intelligence, Hunan University of Technology, Zhuzhou 412007, Chinachengying_02@163.com (Y.C.); m23085400033@stu.hut.edu.cn (L.Z.)

**Keywords:** obstructive sleep apnea syndrome, snoring signals, OSAHS detection, efficient channel attention mechanism, residual networks, bidirectional gated recirculation unit

## Abstract

Obstructive sleep apnea–hypopnea syndrome (OSAHS) is a prevalent sleep disorder strongly linked to increased cardiovascular and metabolic risk. While prior studies have explored snore-based analysis for OSAHS, they have largely focused on either detection or classification in isolation. Here, we present a two-stage framework that integrates precise snoring event detection with deep learning-based classification. In the first stage, we develop an Adaptive Multi-Feature Fusion Endpoint Detection algorithm (AMFF-ED), which leverages short-time energy, spectral entropy, zero-crossing rate, and spectral centroid to accurately isolate snore segments following spectral subtraction noise reduction. Through adaptive statistical thresholding, joint decision-making, and post-processing, our method achieves a segmentation accuracy of 96.4%. Building upon this, we construct a balanced dataset comprising 6830 normal and 6814 OSAHS-related snore samples, which are transformed into Mel spectrograms and input into ERBG-Net—a hybrid deep neural network combining ECA-enhanced ResNet18 with bidirectional GRUs. This architecture captures both spectral patterns and temporal dynamics of snoring sounds. The experimental results demonstrate a classification accuracy of 95.84% and an F1 score of 94.82% on the test set, highlighting the model’s robust performance and its potential as a foundation for automated, at-home OSAHS screening.

## 1. Introduction

Obstructive sleep apnea–hypopnea syndrome (OSAHS) is a prevalent sleep-related breathing disorder characterized by recurrent episodes of upper airway collapse during sleep, leading to apnea and hypoventilation. These episodes are frequently accompanied by loud snoring, disrupted sleep architecture, intermittent oxygen desaturation, and excessive daytime sleepiness [1]. A growing body of evidence has established strong associations between OSAHS and a spectrum of comorbid conditions, including cardiovascular disease [2], cognitive dysfunction [3], hypertension [4], type 2 diabetes [5], and even sudden cardiac death [6]. Epidemiological data suggest that the prevalence of OSAHS in the adult population ranges from approximately 3% to 7% [7], with significantly higher rates observed in older individuals and those with obesity. Given its wide-ranging health implications and insidious progression, early screening and accurate diagnosis of OSAHS are of critical clinical importance.

At present, the clinical diagnosis of OSAHS predominantly depends on polysomnography (PSG), which evaluates sleep architecture and respiratory abnormalities by monitoring a range of physiological signals, including electroencephalography (EEG), electrooculography (EOG), electrocardiography (ECG), electromyography (EMG), and respiratory airflow (RAF) [8]. Despite its diagnostic reliability, PSG is hindered by high equipment costs, operational complexity, dependence on manual annotation, and significant discomfort for patients, limiting its feasibility for large-scale population screening. Consequently, recent research has increasingly focused on developing low-cost, non-contact alternatives to support OSAHS diagnosis. For instance, Kim et al. proposed a severity classification model based on patients’ respiratory sounds [9], while Volák et al. explored OSAHS identification through image-based analysis of craniofacial features in children [10].

As one of the earliest and most observable clinical manifestations of OSAHS, snoring carries physiological information indicative of upper airway narrowing and vibration patterns [11]. Its acquisition is non-invasive, technically simple, and well-suited to home-based monitoring, making snore-based OSAHS screening an emerging area of research. These approaches typically involve two key steps: automatic detection of snore segments and subsequent classification.

Early detection methods predominantly relied on single-threshold feature extraction techniques—such as spectral entropy [12], autocorrelation [13], and empirical mode decomposition (EMD) [14]—to identify the onset and offset of snoring based on acoustic features like energy or periodicity. To enhance robustness, some studies have introduced adaptive or dual-threshold strategies. For example, Wang et al. [15] and Jiang [16] employed an adaptive root mean square thresholding method, where the absolute amplitude of the denoised signal is used to compute a histogram, and 1.5 times the mode is selected as the detection threshold. Fang et al. [17] proposed a dual-threshold scheme based on short-time energy and zero-crossing rate, further integrating support vector machines for refined segment screening. Nevertheless, these approaches often suffer from limited feature diversity, rigid or empirically defined thresholds, and a lack of postprocessing mechanisms that constrain their generalizability and performance in real-world applications.

In snore sound classification, conventional approaches rely primarily on the extraction of handcrafted audio characteristics, such as the mel-frequency cepstral coefficient (MFCC), spectral entropy, and power ratios, followed by machine learning algorithms, including random forests, logistic regression, and support vector machines (SVMs), to distinguish OSAHS patients from habitual snorers [16,17,18]. For example, Shen et al. [19] proposed a feature fusion algorithm based on Fisher’s criterion in conjunction with an SVM classifier, achieving an accuracy of 95.8%. Cheng et al. [20] combined MFCC, filter banks (Fbanks), short-time energy, and Linear Predictive Coding (LPC) features to train a long short-term memory (LSTM)-based classifier, which reached 95.3% accuracy. In parallel, some studies have explored the transformation of snoring signals into visual representations to leverage deep learning. Li et al. [21], for example, developed a convolutional neural network (CNN)-based recognition model using temporal snore spectrograms, achieving classification accuracy of 92.5%. Although these methods have shown high accuracy, they still face two primary limitations. First, many rely on complex and task-specific feature engineering pipelines. Second, a number of studies neglect the influence of confounding acoustic factors, such as vocal timbre, which can adversely affect the robustness of classification.

To address the limitations of existing approaches, this study proposes a two-stage snore analysis framework for OSAHS screening, integrating an Adaptive Multi-Feature Fusion Endpoint Detection algorithm with a deep learning-based classification network for collaborative modeling.

The key innovations and contributions of this work are as follows:

(1) We introduce the AMFF-ED (Adaptive Multi-Feature Fusion Endpoint Detection) algorithm, which integrates four frame-level acoustic features—short-time energy (E), spectral entropy (H), zero-crossing rate (ZCR), and spectral centroid (C)—within an adaptive statistical thresholding scheme. A multi-feature decision mechanism combined with post-processing techniques, including gap merging and minimum-duration constraints, enables robust and accurate localization of snoring events in realistic hospital acoustic conditions.

(2) Based on data collected from 40 OSAHS patients, we constructed a balanced dataset comprising 6830 normal snores and 6814 OSAHS snores. Each sample was transformed into a Mel spectrogram and fed into a custom-designed deep learning architecture, ERBG-Net. The model integrates efficient channel attention (ECA), an enhanced ResNet18 backbone, and a bidirectional gated recurrent unit (BiGRU), enabling joint modeling of the spectral–spatial characteristics and temporal dynamics of snoring. This hybrid design effectively captures discriminative features across both frequency and time domains.

The experimental results demonstrate that the proposed method achieves 96.4% accuracy in the snore detection task and 95.84% accuracy with an F1 score of 94.82% in the classification task. These results highlight the framework’s robustness and effectiveness, offering a comprehensive and scalable solution for home-based OSAHS screening through snoring analysis.

## 2. Dataset and Preprocessing

### 2.1. PSG-Audio

**Figure 1:**
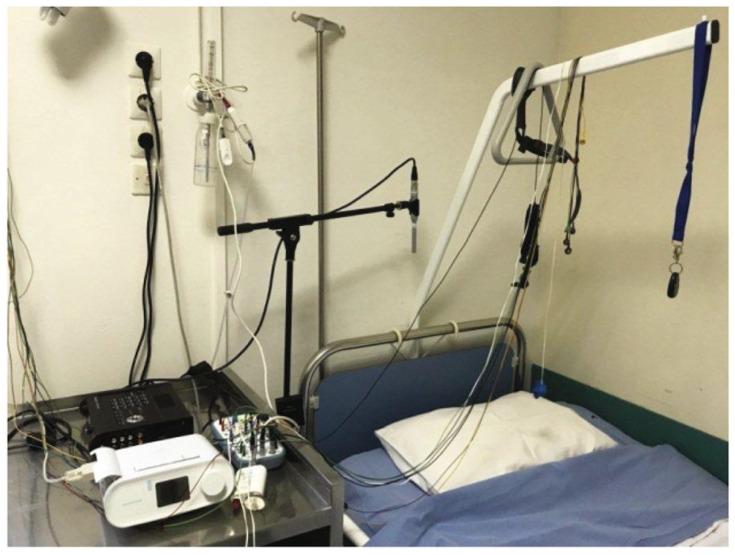
Contact and ambient microphones installed in the sleep study room, along with the multitrack recorder.

The PSG-Audio dataset contains synchronized polysomnographic recordings and high-fidelity audio signals from patients with sleep apnea, making it well-suited for research on sleep-disordered breathing [22]. To collect the data for this dataset, a tracheal microphone was positioned near the patient’s nostrils to capture respiratory sounds, while an ambient microphone—with a 48 kHz sampling rate—was placed approximately 1 m from the bedside to record environmental audio. The experimental setup is illustrated in Figure 1.

The dataset was manually annotated by the medical team at Sismanoglio-Amalia Fleming General Hospital in Athens, based on clinical expertise and polysomnographic monitoring data. Table 1 summarizes all annotated event types, including key sleep apnea-related events such as obstructive apnea, central apnea, and hypoventilation.

To align with the development of non-contact intelligent sleep monitoring technologies, this study focuses on analyzing the audio signals captured by the ambient microphone employed to collect data for the PSG-Audio dataset. Specifically, we selected three hours of nocturnal sleep recordings from 40 patients diagnosed with OSAHS. The demographic and clinical characteristics of the 40 patients are summarized in Table 2. The cohort comprises a male-to-female ratio of 3:1, with ages ranging from 23 to 85 years and a mean age of 57.5 years. The distribution according to Obstructive Sleep Apnea–Hypopnea Syndrome (OSAHS) severity, as measured by the Apnea–Hypopnea Index (AHI), indicated 2 patients with mild, 7 with moderate, and 31 with severe OSAHS. All selected participants had confirmed diagnoses of obstructive sleep apnea to ensure that the classification task focused on distinguishing between different types of snoring events, rather than on inter-subject variability.

### 2.2. Preprocessing

The preprocessing pipeline comprises three key steps: selection of snoring-containing audio segments, spectral subtraction-based denoising, and frame-level windowing.

Using expert annotations of respiratory events, we extracted two distinct categories of audio segments from the original nocturnal recordings: pathological snores associated with OSAHS events, and normal snores with no respiratory abnormalities. These samples served as the foundational input for downstream feature extraction and model training.

To accommodate the inherently low signal-to-noise ratio of nighttime recordings and enhance the robustness of snore analysis, we employed the spectral subtraction method for noise reduction. This technique estimates the average background noise spectrum and subtracts it from the amplitude spectrum of each audio frame, thereby attenuating stationary background interference while preserving the salient components of snoring. The core computation of spectral subtraction is given by the following formulation:

(1) $$|\hat{X}\left(k,l\right)|=\max(|Y\left(k,l\right)|-|\hat{N}\left(k\right)\left|,0\right)$$

where |Y(k,l)| denotes the observed magnitude spectrum of the *l*-th frame at frequency bin *k*; $|\hat{N}\left(k\right)|$ represents the estimated average magnitude spectrum of the background noise; and $|\hat{X}\left(k,l\right)|$ is the estimated clean spectrum of the target signal (i.e., the snoring component) after noise reduction.

Subsequently, the denoised audio signal was segmented into overlapping frames using a sliding-window approach. A frame length of 25 ms and a frame shift of 10 ms were employed to balance time and frequency resolution. To mitigate spectral leakage and ensure temporal smoothness, each frame was multiplied by a Hamming window. The resulting windowed signal for the *i*-th frame is defined as

(2) $$x_{i}\left(n\right)=x\left(n+iH\right)\cdot w\left(n\right),\;0\leq n<N$$

where x(n) is the original time-domain signal, *H* is the frame shift, *N* is the frame length, and w(n) denotes the window function.

## 3. Methods

This study aims to distinguish between normal snoring and OSAHS snoring by analyzing nocturnal sleep recordings. The critical initial step involves accurate extraction of snoring segments, followed by the development of a classification model to differentiate between normal snoring and OSAHS snoring. The methodology is structured into three key stages: multi-feature fusion for precise endpoint detection, Mel spectrogram feature extraction, and the design of the ERBG-Net classification model.

### 3.1. AMFF-ED

**Figure 2:**
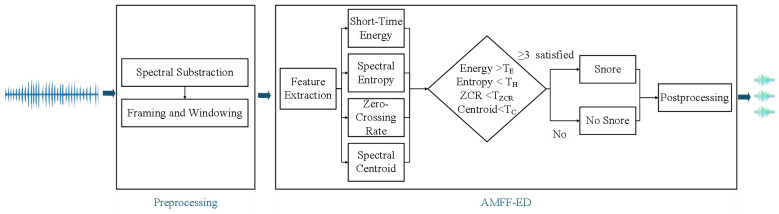
Overview of the preprocessing and adaptive endpoint detection pipeline for snoring signal segmentation.

To precisely detect the onset and offset of snoring events, we propose an Adaptive Multi-Feature Fusion Endpoint Detection (AMFF-ED) algorithm. This method integrates four frame-level acoustic features—energy intensity, spectral flatness, spectral centroid, and waveform smoothness—to capture the multidimensional characteristics of snoring. By fusing these complementary features, AMFF-ED provides a robust and comprehensive representation of snoring signals, encompassing energy dynamics, spectral structure, temporal waveform variation, and frequency content. The complete workflow for preprocessing and snoring endpoint detection is illustrated in Figure 2. The computational framework of AMFF-ED is delineated in detail as follows:

We begin by applying a sliding window of 2 min, with both the window length and step size fixed at 2 min. For the *i*-th frame signal $x_{i}\left(n\right)$ within each window, the following acoustic features are systematically extracted:

(1) Short-time energy $E_{i}$, defined as

(3) $$E_{i}=\sum_{n=0}^{N-1}x_{i}{\left(n\right)}^{2}$$

where *N* denotes the total number of sampling points contained in a single frame.

(2) Spectral entropy $H_{i}$, defined as

(4) $$H_{i}=-\sum_{k=1}^{K}P_{i}\left(k\right)\log\left(P_{i}\left(k\right)+\epsilon \right),\;P_{i}\left(k\right)=\frac{|X_{i}{\left(k\right)|}^{2}}{\sum_{k}{\left|X_{i}\left(k\right)\right|}^{2}}$$

where $P_{i}\left(k\right)$ denotes the normalized power spectrum of the *i*-th frame at frequency index *k*; $|X_{i}{\left(k\right)|}^{2}$ represents the squared spectral magnitude; ϵ is a small constant introduced to avoid numerical singularities in the logarithm; and *k* specifies the total number of frequency bins.

(3) Zero-crossing rate ${\mathrm{ZCR}}_{i}$, defined as

(5) $${\mathrm{ZCR}}_{i}=\frac{1}{2N}\sum_{n=1}^{N}\left|\mathrm{sgn}\left(x_{i}\left(n\right)\right)-\mathrm{sgn}\left(x_{i}\left(n-1\right)\right)\right|$$

where sgn(·) is the sign function that determines the polarity of each sample; $x_{i}\left(n\right)$ denotes the *n*-th sample of the *i*-th frame; and *N* is the number of samples in the frame.

(4) Spectral centroid $C_{i}$, defined as

(6) $$C_{i}=\frac{\sum_{f}f\cdot \left|X_{i}\left(f\right)\right|}{\sum_{f}\left|X_{i}\left(f\right)\right|}$$

where *f* denotes frequency, and $|X_{i}\left(f\right)|$ corresponds to the spectral magnitude of the *i*-th frame at frequency *f*.

Subsequently, statistical thresholds $T_{E}$, $T_{H}$, $T_{\mathrm{ZCR}}$, and $T_{C}$ are computed for all features within each sliding window. Relative to non-snoring segments, snoring frames generally exhibit elevated energy, spectral concentration in the low-to-mid frequency bands, a more compact spectral structure, and smoother waveform profiles. Accordingly, $T_{E}$ is defined as the 10th percentile of the energy distribution, and $T_{C}$ as the 75th percentile of the spectral centroid, whereas $T_{\mathrm{ZCR}}$ and $T_{H}$ are set to “mean + half the standard deviation” to adaptively capture intra-window variability. The formal thresholding rules are given as follows:

(7) $$\begin{array}{cc} T_{E} & ={\mathrm{Percentile}}_{10}\left(E\right)\\ T_{H} & =\mu_{H}+0.5\cdot \sigma_{H}\\ T_{\mathrm{ZCR}} & =\mu_{\mathrm{ZCR}}+0.5\cdot \sigma_{\mathrm{ZCR}}\\ T_{C} & ={\mathrm{Percentile}}_{75}\left(C\right) \end{array}$$

where μ and σ denote the mean and standard deviation of the corresponding feature within the sliding window, respectively.

Each frame is subsequently classified according to the decision function *S*. A frame is identified as a snoring frame if $S_{i}\geq 3$. The decision function is formulated as

(8) $$S_{i}=\sum_{\phi \in \{E,H,Z,C\}}I\left(\phi_{i}\;\mathrm{satisfies}\;\mathrm{threshold}\;\mathrm{condition}\right)$$

To ensure the temporal continuity and accuracy of the detected snoring segments, two post-processing strategies are employed: (1) gap merging, where adjacent snoring segments separated by intervals shorter than $\Delta t_{\mathrm{gap}}=0.3\;s$ are merged into a single continuous segment; (2) shortest-segment filtering, where segments with durations shorter than $\Delta t_{\min}=0.3\;s$ are discarded, ensuring that only physiologically meaningful events are retained.

Finally, AMFF-ED outputs a high-confidence snoring time index $\left[t_{start},t_{end}\right]$, which provides an accurate basis for subsequent snoring segmentation.

### 3.2. Mel Spectrogram

Snoring is a non-stationary physiological audio signal characterized by pronounced nonlinearity and temporal variability. Compared to commonly employed audio features such as MFCC, spectral entropy, and power ratio, the Mel spectrogram offers an optimal balance between time and frequency resolution, enabling more effective preservation of the signal’s energy distribution patterns. Unlike traditional spectral representations, the Mel spectrogram applies frequency mapping to the Mel scale via short-time Fourier transform (STFT), aligning more closely with human auditory perception. This transformation enhances the representation of key frequency components, as described by the following frequency mapping relationship:

(9) $$m\left(f\right)=2595\times \log_{10}\left(1+\frac{f}{100}\right)$$

where m(f) denotes the Mel frequency and *f* represents the linear frequency.

### 3.3. ERBG-Net

**Figure 3:**
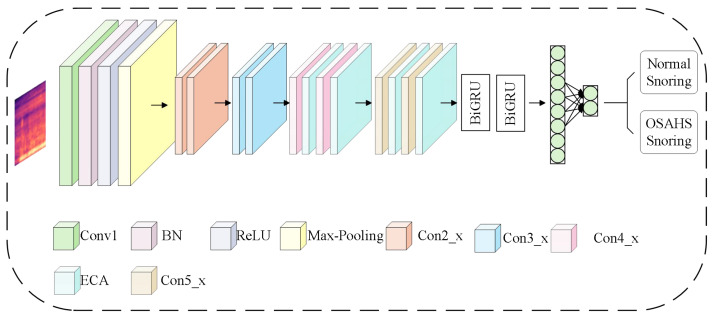
ERBG-Net model architecture.

ERBG-Net is a novel hybrid architecture—efficient channel attention–ResNet–bidirectional gated recurrent unit—designed for automatic classification of snoring signals. The overall model architecture is illustrated in Figure 3. ERBG-Net integrates ResNet18 and BiGRU modules, enhanced by the ECA mechanism. Multilayer convolutional operations and residual connections within ResNet18 facilitate robust extraction of local spatial features from snoring spectrograms. The ECA module adaptively emphasizes critical frequency bands, improving feature representation in the spectral domain. Subsequently, the BiGRU module captures latent rhythmic patterns and temporal dependencies inherent in snoring sequences. Together, these components synergistically bolster the model’s discriminative power across both spatial and temporal dimensions, enabling more accurate snoring classification.

#### 3.3.1. ResNet18 Enhanced with ECA

To effectively extract discriminative spatial features from snoring Mel spectrograms, this study employs ResNet18 as the backbone feature extractor. Unlike conventional shallow convolutional networks, ResNet18 addresses the vanishing gradient and degradation problems commonly encountered in deep network training by introducing residual connections [23]. These connections enable stable optimization of deeper architectures while preserving strong representational capacity, thereby enhancing the model’s ability to capture complex patterns inherent in spectro-temporal snoring signals.

**Figure 4:**
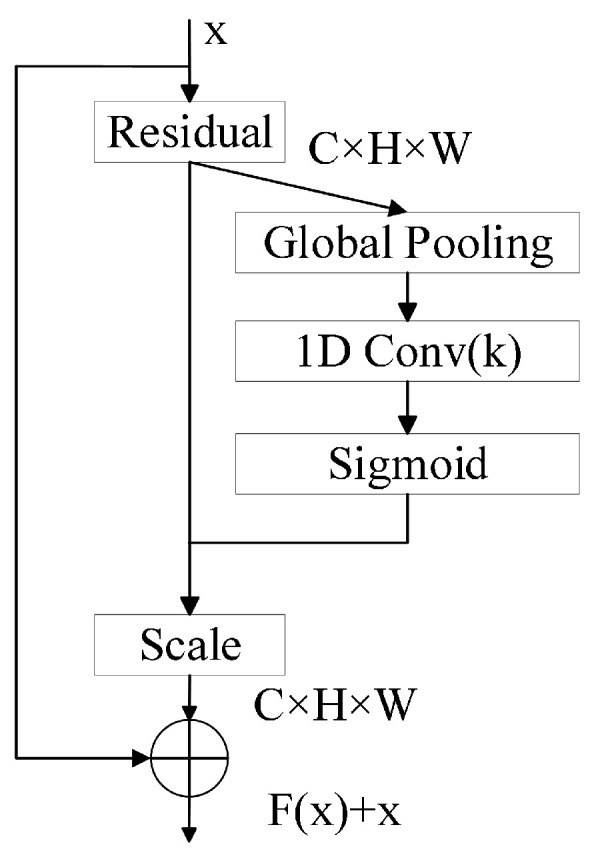
Structure of the improved residual module.

However, the original ResNet18 architecture lacks the capacity to effectively model inter-channel dependencies when processing two-dimensional inputs such as Mel spectrograms, which often exhibit pronounced local variations. This limitation hinders the network’s ability to fully leverage discriminative feature responses from key regions in snoring spectrograms. To address this, the ECA module is incorporated after each convolutional block in the Conv4_x and Conv5_x stages of ResNet18, resulting in an enhanced architecture referred to as ECA-ResNet18. The ECA module enables lightweight channel attention modeling by employing parameter-free 1D convolution without dimensionality reduction, thereby enhancing the network’s ability to focus on salient channels while avoiding significant computational overhead. This modification improves the selectivity and expressiveness of the extracted features. The structure of the improved residual module is illustrated in Figure 4.

The ECA module employs one-dimensional convolution to model local interactions along the channel dimension, thereby avoiding the information loss typically introduced by dimensionality reduction operations. By capturing short-range dependencies between adjacent channels, the module adaptively recalibrates channel-wise feature responses, enhancing sensitivity to task-relevant information. Compared to the traditional Squeeze-and-Excitation (SE) mechanism, ECA achieves improved generalization capability with significantly lower parameter overhead [24]. The computational process of the ECA module is as follows:

Global average pooling is first applied to aggregate spatial information, yielding a single representative value for each channel that captures its global context:

(10) $$y_{i}=\frac{1}{H\times W}\sum_{a=1}^{H}\sum_{b=1}^{W}x_{i}\left(a,b\right)$$

where $x_{i}\left(a,b\right)$ denotes the pixel value at spatial location (a,b) on the *i*-th channel, and $y_{i}$ represents the global average pooling result for channel *i*.

An adaptive one-dimensional convolutional kernel is employed to control the effective receptive field across channels, enabling the module to flexibly capture inter-channel dependencies within a local neighborhood. The kernel size is dynamically determined based on the total number of channels, thereby adapting the receptive field to the complexity of the input feature map.

(11) $$k={\left|\frac{\log_{2}C}{\gamma }+b\right|}_{\mathrm{odd}}$$

where *C* is the number of channels, γ and *b* are hyperparameters, and ${\left|\;\cdot \;\right|}_{\mathrm{odd}}$ ensures that the kernel size is rounded to the nearest odd integer.

A local one-dimensional convolution is applied to model the inter-channel relationships and to generate the corresponding attention weights. The output is passed through a sigmoid activation function σ(·) to ensure the weights are constrained between 0 and 1. Formally,

(12) $$w=\sigma \left(\mathrm{Conv}1D(y)\right)$$

where Conv1D denotes the one-dimensional convolution operation, and *y* is the vector of global descriptors obtained via global average pooling.

The final channel-wise recalibrated output is obtained by reweighting the original feature map:

(13) $${\hat{x}}_{i}=w_{i}\cdot x_{i}$$

where $w_{i}$ is the learned attention weight for the *i*-th channel, and ${\hat{x}}_{i}$ denotes the refined output feature map with enhanced task-relevant responses.

#### 3.3.2. Bidirectional Gated Recurrent Unit

To capture the temporal dynamics inherent in snoring signals, the two-dimensional feature maps extracted by ECA-ResNet18 are unfolded along the time axis and transformed into sequential representations. These sequence features are then fed into a BiGRU network. The GRU architecture introduces an update gate and a reset gate, along with a candidate hidden state computation, which collectively mitigate the vanishing-gradient problem commonly encountered in long-sequence modeling [25]. By processing the sequence in both the forward and backward directions, the BiGRU further enhances the network’s ability to capture bidirectional temporal dependencies, enabling more accurate modeling of rhythmic and context-dependent patterns in snoring signals.

Building upon the GRU framework, the BiGRU incorporates both forward and backward information flows, allowing the model to integrate historical and future temporal contexts [26]. This bidirectional processing significantly enhances the network’s capacity to represent temporal dependencies, particularly in non-stationary signals such as snoring. The computational process of BiGRU at time step *t* is formulated as

(14) $$\vec{h_{t}}=\mathrm{GRU}\left(x_{t},{\vec{h}}_{t-1};W_{t},b_{t}\right)$$

(15) $$\overset{\leftarrow }{h_{t}}=\mathrm{GRU}\left(x_{t},{\overset{\leftarrow }{h}}_{t+1};V_{t},b_{t}\right)$$

(16) $$h_{t}=\left[\vec{h_{t}};\overset{\leftarrow }{h_{t}}\right]$$

where $x_{t}$ denotes the input feature at time step *t*; $\vec{h_{t}}$ represents the hidden state output of the forward GRU at time *t*; $\overset{\leftarrow }{h_{t}}$ represents the hidden state output of the backward GRU at time *t*; $W_{t}$ and $V_{t}$ denote the weight parameters of the forward and backward GRU units at time *t*, respectively; $b_{t}$ is the bias term associated with the GRU hidden states; and $h_{t}$ is the concatenated bidirectional representation, integrating information from both the forward and backward passes.

## 4. Experimental Results

### 4.1. Results of Snoring Detection Experiments

The experimental dataset employed in this study was acquired in a clinical hospital environment, where multiple devices operated concurrently during recording sessions. Consequently, patient sleep recordings inevitably contained persistent device-related electrical noise, as well as intermittent conversational speech, door-closing sounds, and call-bell signals, representing typical environmental interferences. To further emulate a complex and representative acoustic scenario, and in light of prior studies demonstrating the significant disruptive effects of conversational background noise on speech and respiration detection tasks, additional low-level conversational background noise was superimposed onto the original recordings. This constituted an additional comparative experimental condition. Accordingly, four experimental configurations were established:

(1) AMFF-ED + original recordings;
(2) AMFF-ED + noise-reduced recordings;
(3) Short-time energy and ZCR + noise-reduced recordings;
(4) AMFF-ED + original recordings with low-level conversational background noise.

**Figure 5:**
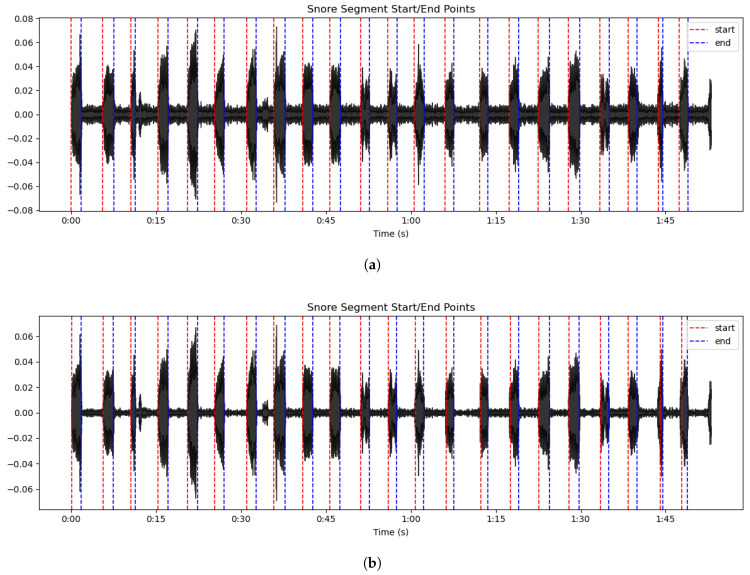

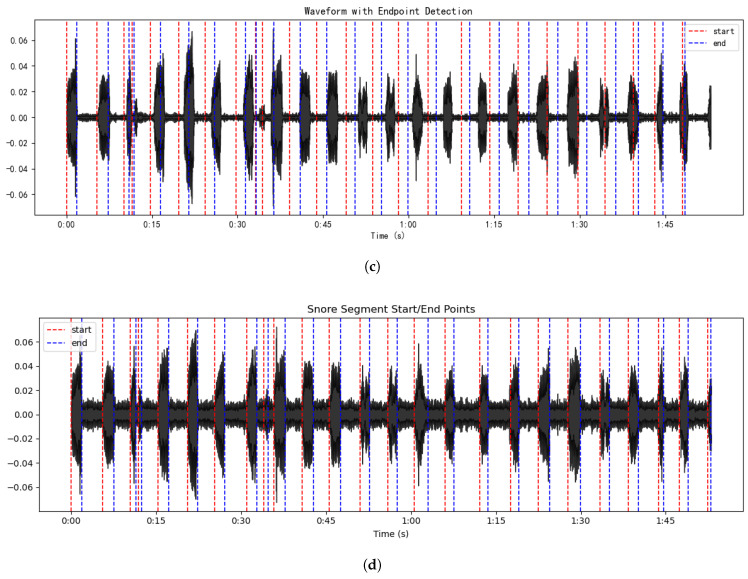
Endpoint detection performance across different acoustic conditions. (**a**) AMFF-ED + original recordings; (**b**) AMFF-ED + noise-reduced recordings; (**c**) Short-time energy and ZCR + noise-reduced recordings; (**d**) AMFF-ED + original recordings with low-level conversational background noise.

The detection results are illustrated in Figure 5. As shown in Figure 5a, AMFF-ED accurately delineates the onset and offset of snoring events in the original recordings while effectively suppressing minor respiratory artifacts; its performance on noise-reduced recordings (Figure 5b) was largely consistent. In contrast, the short-time energy and ZCR method exhibited inferior performance on the same noise-reduced recordings (Figure 5c): although partial snoring events were detected, the onset and offset boundaries displayed systematic deviations, detection completeness was compromised, and background sounds were frequently misclassified as snoring. Notably, Figure 5d demonstrates that, even in the presence of low-level conversational background noise, AMFF-ED maintains accurate and robust detection of snoring onsets and offsets, with only a minimal number of respiratory sounds misclassified and no significant missed detections observed.

To quantitatively evaluate the algorithm across the entire dataset, the endpoint detection accuracy was defined as

(17) $$\begin{array}{c} Accuracy=\frac{f_{r}}{f_{a}}\times 100\% \end{array}$$

where $f_{r}$ denotes the number of snoring segments correctly identified in accordance with PSG annotations, and $f_{a}$ represents the total number of snoring segments detected during the nocturnal recordings.

The evaluation utilized one-hour sleep recordings randomly selected from each of 40 participants. The results across the four experimental configurations are summarized in Table 3.

As shown in Table 3, AMFF-ED consistently achieves high detection accuracy across diverse acoustic conditions, peaking at 96.4% on noise-reduced recordings. Even in complex acoustic environments simulating multi-speaker conversations, its accuracy remains high (91.6%), significantly outperforming conventional short-time energy and ZCR methods. These findings demonstrate that AMFF-ED provides reliable and robust snoring endpoint detection in realistic and challenging auditory contexts.

### 4.2. Model Training and Evaluation

We conducted model training using the previously extracted snoring segments to evaluate the classification performance of ERBG-Net.The experiments were conducted on a Windows 11 operating system equipped with an Intel^®^ Core™ i5-10500 CPU, utilizing Python 3.11 and PyTorch 2.3.1 for model implementation and training, with PyCharm 2024.2.1 as the programming environment. The dataset was partitioned into training, validation, and test sets with a 6:2:2 split. Mel spectrograms of size 128 × 128 were extracted from all audio clips and used as input features for the ERBG-Net model.

**Figure 6:**
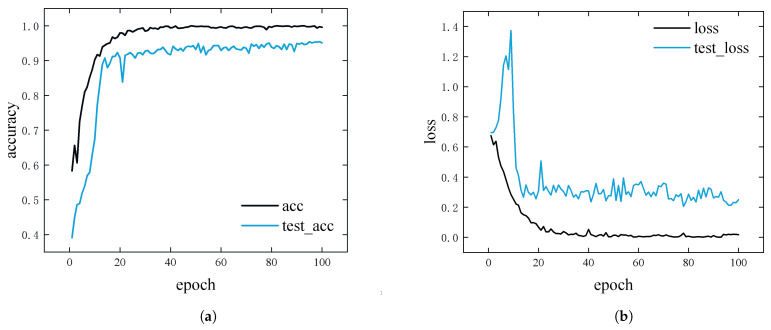
Performance dynamics of the ERBG-Net model during training. (**a**) The classification accuracy trends on the training and validation sets across epochs. (**b**) The corresponding loss function variations during the training process.

Figure 6 depicts the accuracy and loss curves during the training process. The model exhibits a rapid increase in accuracy and a sharp decrease in loss within the first 30 epochs, achieving convergence by approximately the 25th epoch. The final training accuracy reaches 98.5%, with a corresponding loss of 0.005, while the validation set attains an accuracy of 96% and a loss of 0.2. These results demonstrate strong convergence and robust generalization performance of the proposed model.

**Figure 7:**
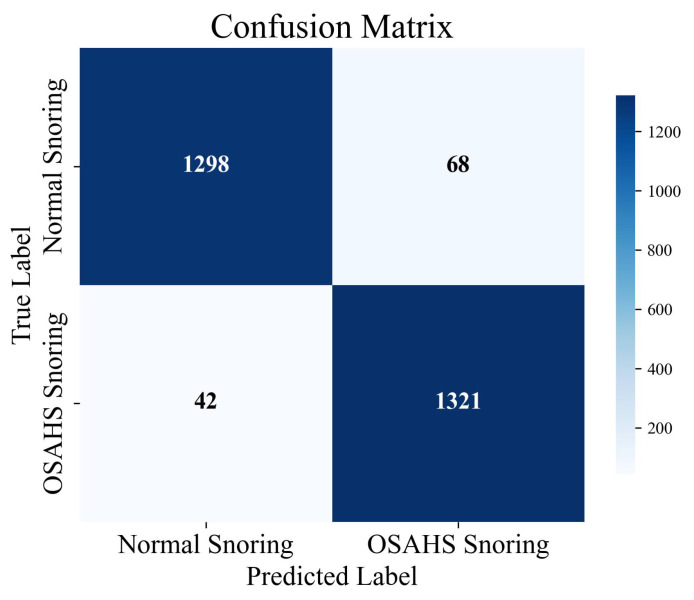
Confusion matrix for snoring classification using ERBG-Net.

Following model training, performance was evaluated on the held-out test set, with the corresponding confusion matrix presented in Figure 7. The ERBG-Net model exhibited high classification accuracy, particularly in recognizing OSAHS-related snoring. However, some misclassifications occurred—specifically, 69 normal-snoring segments were incorrectly identified as OSAHS snoring. This misjudgment may stem from inter-individual variability in snoring characteristics, which can obscure the distinction between normal and pathological snoring patterns, thereby challenging the model’s discriminative capacity.

Despite these minor errors, ERBG-Net demonstrated strong overall classification performance on the dataset, effectively distinguishing between normal and OSAHS snoring. These results highlight its potential as a reliable tool for supporting OSAHS diagnosis through non-invasive, snoring-based analysis.

### 4.3. Ablation Experiment

To comprehensively evaluate the effectiveness of the proposed ERBG-Net in classifying OSAHS-related snoring events, a series of ablation experiments were conducted to examine the individual contributions of key architectural components. Four baseline models were constructed for comparison: ResNet18, ResNet18-BiGRU, ECA-ResNet18, and ECA-ResNet34-BiGRU.

As summarized in Table 4, ERBG-Net achieved a classification accuracy of 95.84%, outperforming all baseline models. Specifically, it exceeded the performance of ResNet18-BiGRU and ECA-ResNet18 by 2.69% and 2.77%, respectively, demonstrating the synergistic effect of combining the ECA mechanism with BiGRU-based temporal modeling.

Furthermore, compared with the ECA-ResNet34-BiGRU model, ERBG-Net achieved a 1.08% higher accuracy. This indicates that under the same ECA and BiGRU framework, adopting the lighter ResNet18 backbone produces a more compact model while maintaining, or slightly improving, classification performance.

Collectively, these results demonstrate the contribution of each module to the overall framework and provide supporting evidence for the robustness and effectiveness of ERBG-Net.

### 4.4. Comparative Experiment

To further assess the efficacy of Mel-frequency cepstrograms in representing snoring-related acoustic features, this study conducted a multi-group comparative experiment employing ERBG-Net as a unified classification framework. The objective was to systematically evaluate the impact of different input features on model performance. Three widely used acoustic representations were selected for comparison: mel-frequency cepstral coefficients combined with the linearly predicted cepstral coefficients (MFCC_LPCC), constant-Q Transform (CQT) time–frequency maps, and conventional sound spectrograms.

**Figure 8:**
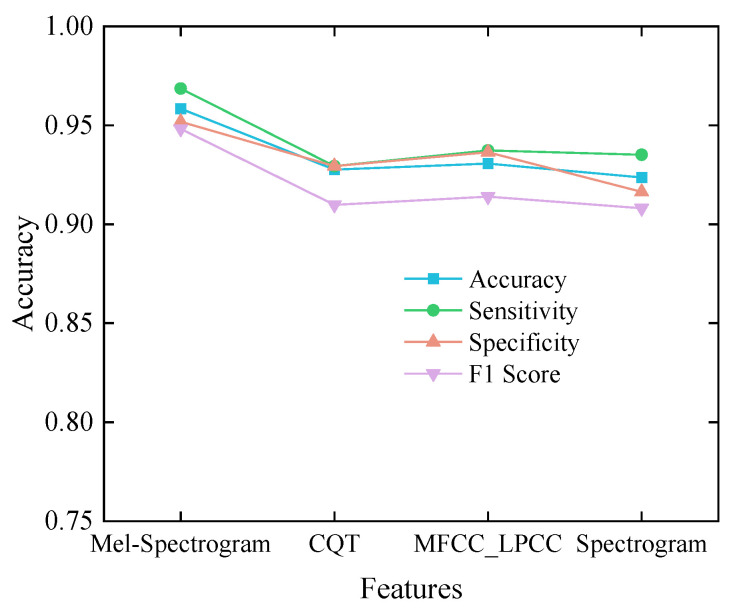
Experimental results of feature comparison.

As illustrated in Figure 8, the Mel spectrogram consistently outperforms the other feature representations across all evaluation metrics. Specifically, it yields the highest classification accuracy of 95.84%, outperforming MFCC_LPCC, CQT, and spectrograms by 2.77, 3.08, and 3.47 percentage points, respectively. Moreover, the Mel spectrogram achieves an F1 score of 94.82%, further demonstrating its superior capacity to capture the intricate time–frequency characteristics of snoring sounds and to enhance the overall classification performance of the model.

## 5. Discussion

This study systematically compares the proposed approach with existing research on OSAHS diagnosis using sleep audio recordings, considering detection strategies, feature representations, model architectures, classification tasks, and accuracy (Table 5). Most prior studies adopt a two-stage framework—snore detection followed by classification—yet the detection stage often relies on conventional speech processing techniques or simplified heuristics, with limited optimization for the acoustic characteristics of snoring.

In detection methods, some studies directly employ speech endpoint detection or unsupervised clustering. For instance, the spectrogram boundary factor-based approach by Shen et al. [18], though effective for speech, exhibits clear limitations when applied to snore signals. Li et al. [21] combined V-Box segmentation, 500 Hz sub-band energy, Principal Component Analysis (PCA) for dimensionality reduction, and Fuzzy C-Means (FCM) clustering, achieving relatively high accuracy but with features constrained to a narrow frequency band and lacking temporal descriptors. Ding [27] and Song [28] used an adaptive effective-value threshold method, but its reliance on a single preset threshold and validation on small samples restricts the generalizability of their results. Several works [20,29] further depend on manual inspection or PSG-assisted annotation; while accurate, such strategies are inherently non-scalable.

**Table 5:** Comparison of related methods for OSAHS diagnosis using sleep audio recordings.

Author	Year	Subjects	Detection	Features	Model	Classification	Accuracy
Shen [18]	2020	32	Spectrogram boundary factor	MFCC	LSTM	Normal vs. abnormal snore	87%
Cheng [20]	2022	43	Endpoint detection + manual check	MFCC, Fbanks, energy, LPC	LSTM	Normal vs. abnormal snore	95.3%
Sillaparaya [29]	2022	5	Manual PSG-based segmentation	Mean MFCC	FC	Normal/apnea–hypopnea snore/non-snore	85.3%
Castillo [30]	2022	25	Not Applicable	Spectrogram	CNN	Apnea vs. non-apnea sounds	88.5%
Li [21]	2023	124	Unsupervised clustering	VG features	2D-CNN	Normal vs. OSAHS snore	92.5%
Song [28]	2023	40	Adaptive thresholding	MFCC, PLP, BSF, PR800, etc.	XGBoost + CNN + ResNet18	Normal vs. abnormal snore	83.4%
Ding [27]	2024	120	Adaptive thresholding	MFCC, VGG16, PANN features	XGBoost + KNN/RF	Normal vs. OSAHS snore	100%
Ours	2025	40	AMFF-ED	Mel-spectrogram	ERBG-Net	Normal vs. OSAHS snore	95.8%

Abbreviations not defined in the main text are provided in the Abbreviations section of this manuscript.

In contrast, our proposed AMFF-ED algorithm demonstrates superior adaptability and robustness in snore detection. First, by integrating multi-dimensional features—short-time energy, spectral entropy, zero-crossing rate, and spectral centroid—it captures snore attributes across the energy, temporal, and spectral domains, overcoming the limitations of single-feature approaches. Second, adaptive thresholds based on sliding-window statistics and quantiles, together with gap merging and minimum-segment filtering, enhance detection continuity and stability. Third, in recordings from 40 subjects, AMFF-ED achieved accuracies of 93.8% and 96.4% under raw and denoised conditions, respectively—significantly surpassing the conventional energy + ZCR method (78.3%)—indicating stronger robustness and generalization in real-world applications.

For snore classification, deep learning methods combined with acoustic features have gained traction, yet many suffer from redundant feature design, class imbalance, or insufficient generalizability. For example, Shen [18] employed MFCC with LSTM on data from 32 subjects, achieving 87% accuracy and demonstrating the value of temporal modeling. Cheng [20] fused MFCC, filter-bank, short-time energy, and Linear Predictive Coding (LPC) features with LSTM, raising the accuracy to 95.3% but at the cost of a complex and redundant extraction pipeline. Sillaparaya’s [29] approach, limited to five subjects and with disrupted temporal structures, struggled to capture dynamic patterns. Castillo [30] applied spectrogram-based CNNs for apnea detection (88.5% accuracy), but severe imbalance yielded a precision of only 13%. Ding [27] reported 100% accuracy, yet the dataset was heavily skewed (10 normal vs. 110 OSAHS cases), undermining the generalization of their results. Li’s [21] visibility graph (VG) + CNN method achieved 92.5% accuracy and showed innovation in feature construction, though at the expense of computational efficiency and interpretability. Song [28] fused 17 acoustic features with multiple classifiers but achieved only 83.44% accuracy, lower than several single models, suggesting ineffective ensemble complementarity.

To address these limitations, we developed ERBG-Net, which demonstrated clear advantages in classification. First, it leverages AMFF-ED-derived Mel spectrograms as inputs—a representation that is both computationally efficient and physically interpretable. Second, by integrating channel attention–enhanced ResNet18 with BiGRU, the model jointly captures spatial and temporal dependencies, effectively modeling long-term respiratory dynamics while emphasizing salient time–frequency cues. Third, on a balanced dataset with approximately equal proportions of normal and OSAHS snores, ERBG-Net achieved 95.84% accuracy, underscoring its robustness and generalization capacity.

Taken together, the proposed framework advances both detection and classification. AMFF-ED introduces a robust, adaptive, and feature-rich detection strategy, while ERBG-Net enhances classification through synergistic spatiotemporal modeling. Their integration achieves an optimal balance between accuracy, efficiency, and scalability, offering a practical and impactful pathway for the screening and auxiliary diagnosis of snore-based OSAHS.

## 6. Conclusions

This study presents a two-stage framework for the detection and classification of snoring signals, with the aim of facilitating non-invasive screening of obstructive sleep apnea–hypopnea syndrome (OSAHS). First, an Adaptive Multi-Feature Fusion Endpoint Detection (AMFF-ED) algorithm is proposed, which integrates multiple acoustic features to accurately segment snoring episodes. The AMFF-ED method achieves an endpoint detection accuracy of 96.4%. Its design incorporates a multi-feature adjudication mechanism and post-processing strategy, significantly reducing misdetection rates and providing a reliable foundation for subsequent classification.

Building upon this, a dataset comprising normal and OSAHS-related snoring segments is constructed and transformed into Mel spectrogram representations. These are used as inputs to a novel deep learning model, ERBG-Net, which combines a channel attention–enhanced ResNet18 architecture with a BiGRU network. This hybrid design enables complementary modeling of spatial and temporal features, resulting in a test set classification accuracy of 95.84% and an F1 score of 94.82%.

While the proposed framework demonstrates strong classification performance, further improvements are warranted in terms of generalizability and real-time inference capability. Future work will explore more granular OSAHS severity classification, incorporate multimodal physiological signals to enrich feature representation, and pursue model compression and optimization techniques aimed at low-power, lightweight deployment. These advancements will enhance the feasibility of applying the system in wearable devices and home-based monitoring platforms, contributing to earlier detection and personalized health management of OSAHS.

## Figures and Tables

**Table 1:** Annotated event families and corresponding types of snoring events.

Event Family	Specific Types of Snoring Events
Respiratory	Obstructive Apnea/Central Apnea/Mixed Apnea/Hypopnea/Cheyne Stokes Respiration/Periodic Respiration/Respiratory Effort-Related Arousal (RERA)
Neurological	Alternating Leg Muscle Activation/Hypnagogic Foot Tremor/Excessive Fragmentary Myoclonus/Leg Movement/Rhythmic Movement Disorder
Nasal	Snore
Cardiac	Bradycardia/Tachycardia/Long RR/Ptt Drop/Heart Rate Drop/Heart Rate Rise/Asystole/Sinus Tachycardia/Narrow Complex Tachycardia/Wide Complex Tachycardia/Atrial Fibrillation
$SpO_{2}$	Relative Desaturation/Absolute Desaturation

**Table 2:** Demographic and clinical characteristics of study participants.

Parameter	Range/Value
Gender (Male/Female)	3:1
Age Range	23–85 years
Mean Age	57.5 years
AHI Severity Distribution (Mild/Moderate/Severe)	2:7:31

**Table 3:** Accuracy comparison of different endpoint detection methods.

Method	Accuracy (%)
AMFF-ED + original recordings	93.8
AMFF-ED + noise-reduced recordings	96.4
Short-time energy and ZCR + noise-reduced recordings	78.3
AMFF-ED + original recordings with low-level conversational background noise	91.6

**Table 4:** Results of the ablation experiments.

Model	Accuracy	Sensitivity	Specificity	F1 Score
ResNet18	0.9214	0.9294	0.9162	0.9029
ResNet18-BiGRU	0.9315	0.9412	0.9315	0.9195
ECA-Resnet 18	0.9307	0.9176	0.9391	0.9123
ECA-Resnet34-BiGRU	0.9476	0.9412	0.9518	0.9339
ERBG-Net	0.9584	0.9686	0.9518	0.9482

## Data Availability

The data that support the findings of this study are available upon request from the corresponding authors. The data are not publicly available due to ongoing follow-up research.

## Abbreviations

The following abbreviations are used in this manuscript:

AHI	Apnea–Hypopnea Index
AMFF-ED	Adaptive Multi-Feature Fusion Endpoint Detection
BiGRU	Bidirectional Gated Recurrent Unit
BSF	Band Spectral Features
CNN	Convolutional Neural Network
CQT	Constant-Q Transform
DNN	Deep Neural Network
ECA	Efficient Channel Attention
ECG	Electrocardiography
EEG	Electroencephalography
EMD	Empirical Mode Decomposition
EMG	Electromyography
ERBG-Net	Enhanced ResNet–BiGRU Network
FC	Fully Connected
FCM	Fuzzy C-Means
Fbanks	Filter Banks
KNN	k-Nearest Neighbor
LPC	Linear Predictive Coding
LSTM	Long Short-Term Memory
MFCC	Mel-Frequency Cepstral Coefficients
MFCC_LPCC	Mel-Frequency Cepstral Coefficients combined with Linearly Predicted Cepstral Coefficients
OSAHS	Obstructive Sleep Apnea–Hypopnea Syndrome
PCA	Principal Component Analysis
PLP	Perceptual Linear Prediction
PSG	Polysomnography
PR800	Pitch Rhythm at 800 Hz
RAF	Respiratory Airflow
ResNet	Residual Network
RF	Random Forest
RERA	Respiratory Effort-Related Arousal
STFT	Short-Time Fourier Transform
SVM	Support Vector Machine
VG	Visibility Graph
ZCR	Zero-Crossing Rate
